# Transplantation of brown adipose tissue up-regulates miR-99a to ameliorate liver metabolic disorders in diabetic mice by targeting NOX4

**DOI:** 10.1080/21623945.2020.1721970

**Published:** 2020-01-30

**Authors:** Ping Li, Cunxia Fan, Yingying Cai, Shu Fang, Yanmei Zeng, Yudan Zhang, Xiaochun Lin, Hongbin Zhang, Yaoming Xue, Meiping Guan

**Affiliations:** a Department of Endocrinology & Metabolism, Nanfang Hospital, Southern Medical University, Guangzhou, Guangdong, China; b Department of Endocrinology, Affiliated Hospital of Guangdong Medical University, Zhanjiang, Guangdong, China; c Department of Endocrinology and Metabolism, Hainan General Hospital, Haikou, Hainan, China; d Women and Children’s Hospital, School of Medicine, Xiamen University, Xiamen, Fujian, China; e Department of Biomedical Sciences, University of Copenhagen, Copenhagen, Denmark

**Keywords:** Brown adipose tissue, miR-99a, non-alcoholic fatty liver disease, NOX4

## Abstract

Nonalcoholic fatty liver disease (NAFLD), main cause of liver damage, is inextricably linked to diabetes. However, there is no specific means to improve the pathology of fatty liver in diabetic patients. Brown adipose tissue (BAT) is an important endocrine organ that secretes adipokines and microRNAs (miRNAs) involved in systemic metabolic regulation. To investigate the effects of BAT transplantation on liver lipid metabolism in diabetic mice, we transplanted BAT from male donor mice into diabetic mice induced by streptozotocin (STZ) combined with high-fat diet (HFD). At 10 weeks after transplantation, BAT transplantation significantly decreased the blood glucose and lipid, downregulated FAS, CD36, Scd1, ACCα, NOX2, NOX4, TGF-β1, FN and COL-1, up-regulated Nrf2, reversed the pathological changes of liver and increased the circulating miR-99a in diabetic mice. To verify whether circulating miR-99a improves oxidative stress by targeting inhibition of NOX4, we used 0.4mM palmitic acid (PA) to treat the LO2 cells. The expression of NOX4 protein was significantly decreased after transfection with miR-99a mimic, and increased after transfection with miR-99a inhibitor. Luciferase reporter assay confirmed that miR-99a could target NOX4 mRNA. These findings clarify the role of miR-99a and NOX4 in liver beneficial effect of BAT transplantation in diabetic mice.

## Introduction

NAFLD is a chronic liver disease characterized by hepatic steatosis excluding alcohol and other clearly defined liver-damaging factors, including simple steatosis (NAFL), non-alcoholic steatohepatitis (NASH) and related cirrhosis and hepatocellular carcinoma [1]. NAFLD affects approximately 25% of the general population worldwide [2]. The vast majority of NAFLD occurs in people with obesity and its related metabolic abnormalities such as diabetes and dyslipidemia [3,4]. Insulin resistance, excessive lipid deposition in liver, hepatic triglyceride accumulation, oxidative stress and lipid peroxidation play important roles in the pathogenesis of NAFLD [5]. NAFLD can cause serious consequences such as chronic kidney disease (CKD) [6], hepatitis and cirrhosis [7], and further aggravate the metabolic disorder of diabetic patients. However, the current treatment methods are very limited.

Adipose tissue is a large, heterogeneous, pleiotropic, rather complex tissue that exists in almost all of the human body’s cavities. There are white adipose tissue (WAT), BAT and beige adipocytes, whose function depends on the location of the distribution, the cellular composition of the tissue, and the energy state of the body [8]. Unlike the unilocular white adipocytes, the brown adipocytes in BAT and beige adipocytes in WAT are multilocular and contain a large number of mitochondria which are enriched with uncoupling protein 1 (UCP1). WAT stores energy primarily in the form of triglycerides. BAT is mainly distributed in the dorsal interscapular region of rodents [9] and the supraclavicular and cervical regions of humans [10]. In addition to being mainly responsible for heat production, BAT also plays an important role in the energy consumption and the balance of glycolipid metabolism of the human body [11]. Moreover, BAT is an important endocrine organ that secretes a variety of adipokines [12] and miRNAs [13] involved in systemic metabolic regulation, and the latter are involved in the regulation of a variety of biological processes and metabolic homoeostasis, including lipid metabolism, inflammation, oxidative stress, etc. Recent studies have found that transplantation of adipose tissue-derived stem cells (ADSCs) can significantly improve NAFLD and related liver fibrosis and cirrhosis in the rat or mouse model [14–17]. And the study has shown that BAT transplantation can improve glucose tolerance, increased insulin sensitivity, lower body weight, decreased fat mass and a complete reversal of high-fat diet (HFD)–induced insulin resistance in mice [18]. However, there is no research on the improvement of liver lipid metabolism in diabetes with BAT transplantation. In the present study, we investigated whether BAT transplantation can improve liver metabolism in diabetic mice and its possible mechanisms. We found that BAT transplantation can regulate glucolipid metabolism, inhibit liver lipid synthesis, oxidative stress and fibrosis in diabetic mice induced by HFD combined with STZ. The mechanism may be that BAT transplantation increased the circulating level of miR-99a, which improved lipid metabolism, oxidative stress and fibrosis in the liver by negatively regulating the expression of NOX4.

## Materials and methods

### Animal

Thirty-one male healthy C57BL/6J mice aged 5 to 6 weeks were purchased from the Experimental Animal Centre of Guangdong Province. They were housed under a standard 12-h light/12-h dark cycle with 22°C and 60% relative humidity. All animals were fed and watered ad libitum and were fed adaptively for 1 week. The disposal of animals was in accordance with the requirements of the Animal Experimental Ethics Committee of Southern Medical University.

### Preparation of diabetic mice model

Thirty-one male healthy C57BL/6J mice were randomly divided into normal control group (Con) (n = 8) and diabetic group (DM) (n = 23). DM group mice were fed with HFD (D12492; 5.24 kcal/g; 60% kcal from fat, 20% kcal from carbohydrate; Guangdong Medical Laboratory Animal Centre) for 4 weeks and then injected intraperitoneally with STZ (S0130, Sigma; 12 mg/mL STZ dissolved in citrate buffer at pH 4.2 ~ 4.5) of 120 mg/kg body weight. Diabetic mice were diagnosed with random blood glucose ≥13.9 mmol/L (250 mg/dl) after 4 weeks of continuous feeding of HFD [19,20]. Control group mice were fed with chow diet. After 4 weeks, an equal dose of sodium citrate buffer was administered intraperitoneally, and then the chow diet feed was continued for 4 weeks.

### BAT transplantation and specimen collection

Diabetic mice were randomly divided into two groups: BAT transplantation group (DM+TP) (n = 9) and diabetes sham operation group (DM-Con) (n = 9). Firstly, after cervical dislocation, the interscapular BAT (0.2 g) of three-week-old healthy male C57BL/6J mice from the Experimental Animal Centre of Guangdong Province were removed and incubated in sterile PBS at 37°C for 20–30 min. Then, mice in group DM+TP were anaesthetized with intraperitoneal injection of 50 mg/kg body weight pentobarbital sodium and the skin of the dorsal was transversely cut about 0.5 cm. Finally, the donor-derived BAT was transplanted under the skin of mice in group DM+TP. For the mice in group DM-Con, they received a sham operation performed with the same procedure. Monitoring body weight (BW) and random blood glucose (RBG) during the experiment. RBG was postprandial random blood glucose and measured in the blood obtained from the tail vein. Ten weeks after transplantation, mice were sacrificed after anaesthesia with pentobarbital sodium and the blood, liver tissues and BAT were collected.

### Triglyceride and low-density cholesterol content measurement

Serum triglyceride (TG) and low-density cholesterol (LDL-C) were measured using ELISA kits (Nanjing Jiancheng Technology Co., Ltd., Nanjing, China), according to the manufacturer’s instructions.

### Histopathological examination

Liver tissues were fixed in 4% paraformaldehyde for 24 h, embedded in paraffin and cut into sections of 5 μm thickness. Tissue sections were stained with haematoxylin and eosin (H&E). Frozen liver tissues were stained with an Oil Red O staining kit (Beijing Leigen Biotechnology Co., Ltd., Beijing, China) for observing the fatty infiltration. In order to observe the collagen deposition of the liver, Sirius Red staining was performed using a commercial kit (Beijing Leigen Biotechnology Co., Ltd., Beijing, China) according to the protocol.

### Cell culture and in vitro cell model of lipid accumulation

Human normal hepatocyte cell line (LO2) and human embryonic kidney 293 cell line (HEK293) were purchased from the Type Culture Collection of the Chinese Academy of Sciences, Shanghai, China. All cells were incubated at 37°C in a humidified atmosphere containing 5% CO2. All cells were cultured in high glucose (25mM) Dulbecco’s modified Eagle’s medium (DMEM) (Gibco, Carlsbad, CA, USA) containing 10% foetal bovine serum (FBS) (Gibco). The mediums were changed every 24 h. PA powder (Sigma-Aldrich) was dissolved in 0.01 M NaOH. This solution was added to a 20% BSA solution free of fatty acid (Sigma-Aldrich) and the final solution designated as PA. PA was diluted to the specified concentration with DMEM. All cells were grown to 70-80% confluence and starved for 12 h before exposing to PA for 24 h. BSA was used as the control.

### Transfection of plasmid, miRNA mimic and inhibitor

The pENTER-NOX4 plasmid and the empty vector pENTER were purchased from Guangzhou Boxin Biotechnology Co., Ltd. (Guangzhou, China). miR-99a-5p mimics, miR-99a-5p inhibitor, and the corresponding negative controls were purchased from Guangzhou Ruibo Biotechnology Co., Ltd. (Guangzhou, China). Cells were seeded in the 12-well plates with 50-60% confluence and performed the transfection experiment. Plasmid (2μg), mimic, inhibitor or negative controls (50–100 nM) was transfected using Lipofectamine 3000 reagent (Thermo Fisher Scientific, MA, USA) according to the protocol. Cells were collected after 48 h of transfection.

### qRT-PCR analysis

Total RNA of liver tissue and cultured cells was isolated using Trizol reagent. Isolation of BAT and serum miRNA using miRcute miRNA Isolation Kit (TIANGEN, Beijing, China). RNA concentration and purity were determined using an ND-1000 spectrophotometer (Thermo Fisher Scientific). The mRNA was synthesized using reverse transcription reagent (TAKARA, Dalian, China) and the cDNA of miRNA was synthesized using miRcute miRNA cDNA first strand synthesis kit (TIANGEN) according to the manufacturer’s instructions. qRT-PCR analysis was performed using Roche Light Cycler 480II system (Roche, Basle, Switzerland). PCR products were detected using SYBR Primer Script PCR Kit (TIANGEN). Primer sequences are listed in Table 1. The relative expression of the target gene was normalized by β-actin and U6 gene. Relative gene expression was calculated with the 2-ΔΔCt method.

**Table 1.** Primer sequences

Gene	Forward (5ʹ-3ʹ)	Reverse (5ʹ-3ʹ)
β-actin (mouse)	GTCCACCCCGGGGAAGGTGA	AGGCCTCAGACCTGGGCCATT
FAS (mouse)	GGCCAGTGCTATGCTGAGAT	CACTTCTGGAGACATCGCAAAC
CD36	ATGGGCTGTGATCGGAACTG	TTTGCCACGTCATCTGGGTTT
ACCα (mouse)	TATTCGGCTGAAGCTGGTGTAC	TTCTTGCGATACACTCTGGTGC
TGF-β1	TGTGTTGGTTGTAGAGGGCAAGGA	TTTGGAGCCTGGACACACAGTACA
COL-1	GCAGGAGGTTTCGGCTAAGT	GCAACAAAGTCCGCGTATCC
FN	TAGCCCTGTCCAGGAGTTCA	CTGCAAGCCTTCAATAGTCA
Nrf2	CAAACATTCAAGCCGATAGG	CGGCAACTTTATTCTTCCCTCT
NOX4	TGCCTGCTCATTTGGCTGT	CCGGCACATAGGTAAAAGGATG
NOX2	ACCTTACTGGCTGGGATGAA	ATTCTGGTGTTGGGGTGTTG
β-actin (human)	CATGTACGTTGCTATCCAGGC	CTCCTTAATGTCACGCACGAT
PPARγ	GGGATCAGCTCCGTGGATCT	TGCACTTTGGTACTCTTGAAGTT
FAS (human)	ATGTCAGTCACTTGGGCATTA	CATCTGGACCCTCCTACCTCT
ACCα (human)	ATGTCTGGCTTGCACCTAGTA	CCCCAAAGCGAGTAACAAATTCT
SREBP-1c	CGGAACCATCTTGGCAACAGT	CGCTTCTCAATGGCGTTGT
PPARα	CTGGCATTTGTTTCTGTTCTTT	CTCCTCGGTGACTTATCCTGT
CPT-1	TCCAGTTGGCTTATCGTGGTG	TCCAGAGTCCGATTGATTTTTGC
U6	TGGCCCCTGCGCAAGGATG	Universal reverse primer (TIANGEN)
miR-99a-5p	AACCCGUAGAUCCGAUCUUGUG	Universal reverse primer (TIANGEN)

### Western Blot

The protein from liver tissues and LO2 cells was dissolved in RIPA buffer (Beyotime Institute of Biotechnology, Shanghai, China) with protease and phosphatase inhibitors (KGI Biotechnology Co., Ltd., Guangzhou, China). The protein concentration was determined by BCA assay. Equal amounts of protein lysate (30 μg) were separated by 10% SDS-PAGE (BioRad, Hercules, CA, USA), transferred to PVDF membrane (Merck Millipore, MA, USA), blocked in 5% skim milk in TBST for 1 h, probed with the NOX4 (1:1000, Abclone), TGF-β1 (1:500, Abclone), Nrf2 (1:500, Abclone) and β-actin antibody (1:500, Zhongshan Jinqiao Biotechnology Co., Ltd., Beijing, China) at 4°C overnight and then incubated with secondary antibodies conjugated with HRP (LI-COR, Lincoln, NEA) at room temperature for 1 h. Signals were detected with Odyssey near-infrared imaging system (LI-COR) and quantified by Image J software.

### Luciferase reporter assay

Full-length of 3ʹUTR of the NOX4 was amplified by PCR (forward: 5ʹ-CGCCTCCCGGGTTTGCACCACTCTCCTGCCTCAGCCTCCTG-3ʹ; reverse: 5ʹ-ATCATTTTTATTGTCTCAAGAAGAACTTAATAGCAATTAG-3ʹ). The wild-type NOX4 3ʹ-UTR sequences and its mutant-type sequences were inserted into the pmirGLO vector (Guangzhou Boxin Biotechnology Co., Ltd.), respectively. Empty particle vector as control. HEK293 cells were cotransfected with reporter plasmid, and miR-99a mimic or negative control. Luciferase activity was determined by dual-luciferase reporter system (promega) 48 h after transfection.

## Statistical analysis

Data were processed using SPSS 20.0 software and GraphPad software, and the data were expressed as mean ± SEM. Two independent sample means were compared using the Student *t* test. Multiple sample means were compared using one-way ANOVA. P < 0.05 was considered statistically significant.

## Results

### BAT transplantation improved glucolipid metabolism of diabetic mice

**Figure 1.**
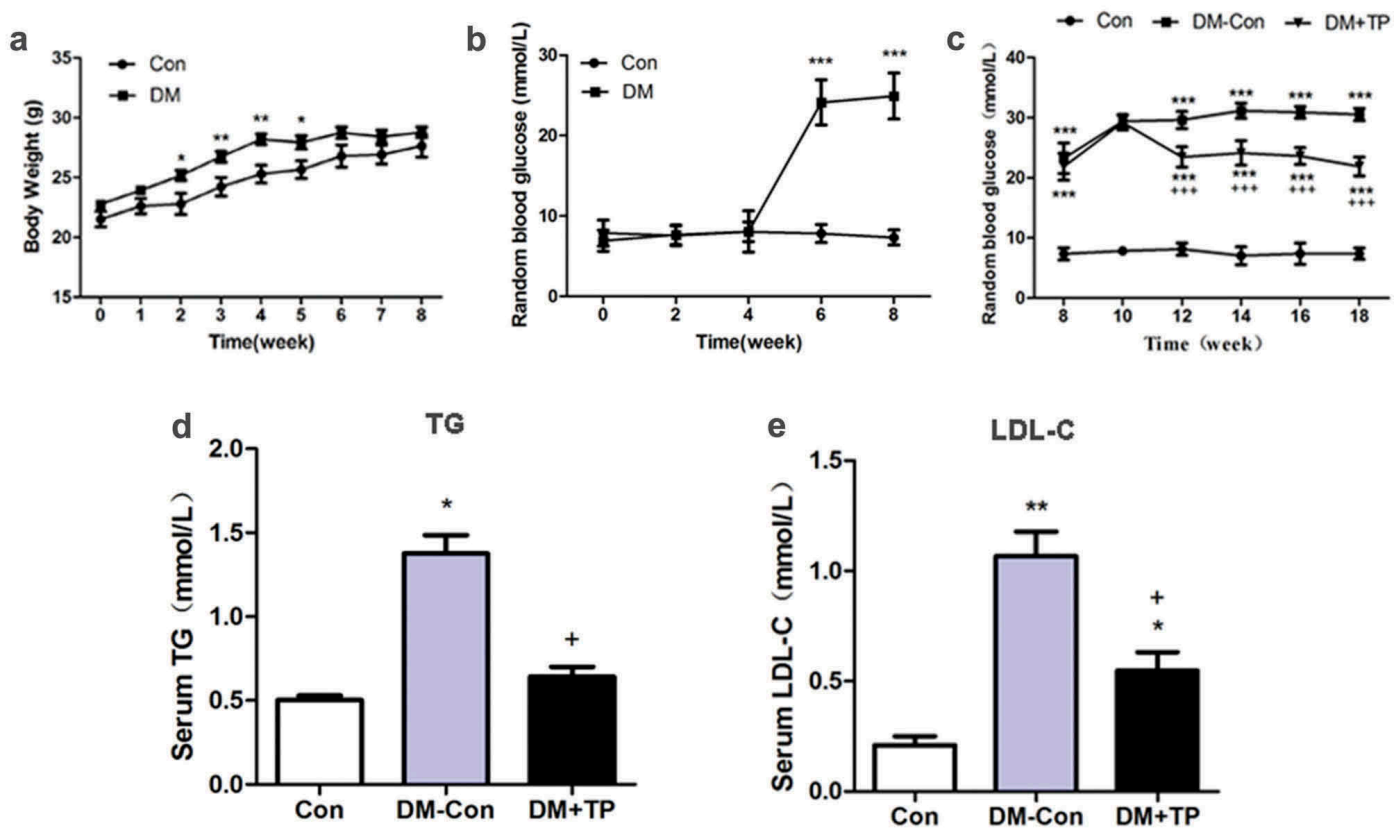
The changes in body weight (a) and random blood glucose (b) during the generation of type 2 diabetic mice (n = 8-23/group). And the changes of RBG (c), serum TG (d) and LDL-C (e) in each groups after BAT transplantation (n = 5-8/group). *P < 0.05 vs Con; **P < 0.01 vs Con; ***P < 0.001 vs Con; ^+^P < 0.05 vs DM-Con; ^+++^P < 0.001 vs DM-Con

During the modelling of diabetic mice, we monitored BW and RBG of all the mice. By 2 weeks after feeding HFD (i.e. week 2), the BW of DM group was significantly higher than that of Control (*P*< 0.05) and the difference was most obvious between week 3 and week 4 (*P*< 0.01). However, after STZ injection (i.e., week 4), there was no significant difference between two groups from week 6 (Figure 1(a)). Before intraperitoneal injection of STZ (i.e., week 4), the RBG of DM group mice was in the normal range. But it was progressively increased after injection of STZ, and there was a significant difference compared with that of Control from the sixth week (*P*< 0.001) (Figure 1(b)). These mean that the model of type 2 diabetic mice was successfully established at week 8 by using HFD and STZ. From 4 weeks after BAT transplantation (i.e., week 12), the RBG of DM+TP group mice was significantly lower than that of DM-Con group (*P*< 0.001), but strikingly, it still significantly higher than that of Con group (*P*< 0.001) (Figure 1(c)).

To investigate the effects of BAT transplantation on blood lipids, we measured the serum TG and LDL-C of the mice in each group. The results showed us that the serum TG and LDL-C in DM-Con group mice were up-regulated significantly compared with those in the Control group. BAT transplantation can down-regulate them significantly compared with those DM-Con group (Figure 1(d,e)).

These data showed that BAT transplantation can improve the glucolipid metabolism of the type 2 diabetic mice.

### BAT transplantation reversed hepatic pathological changes and ameliorated liver metabolism in diabetic mice

**Figure 2.**
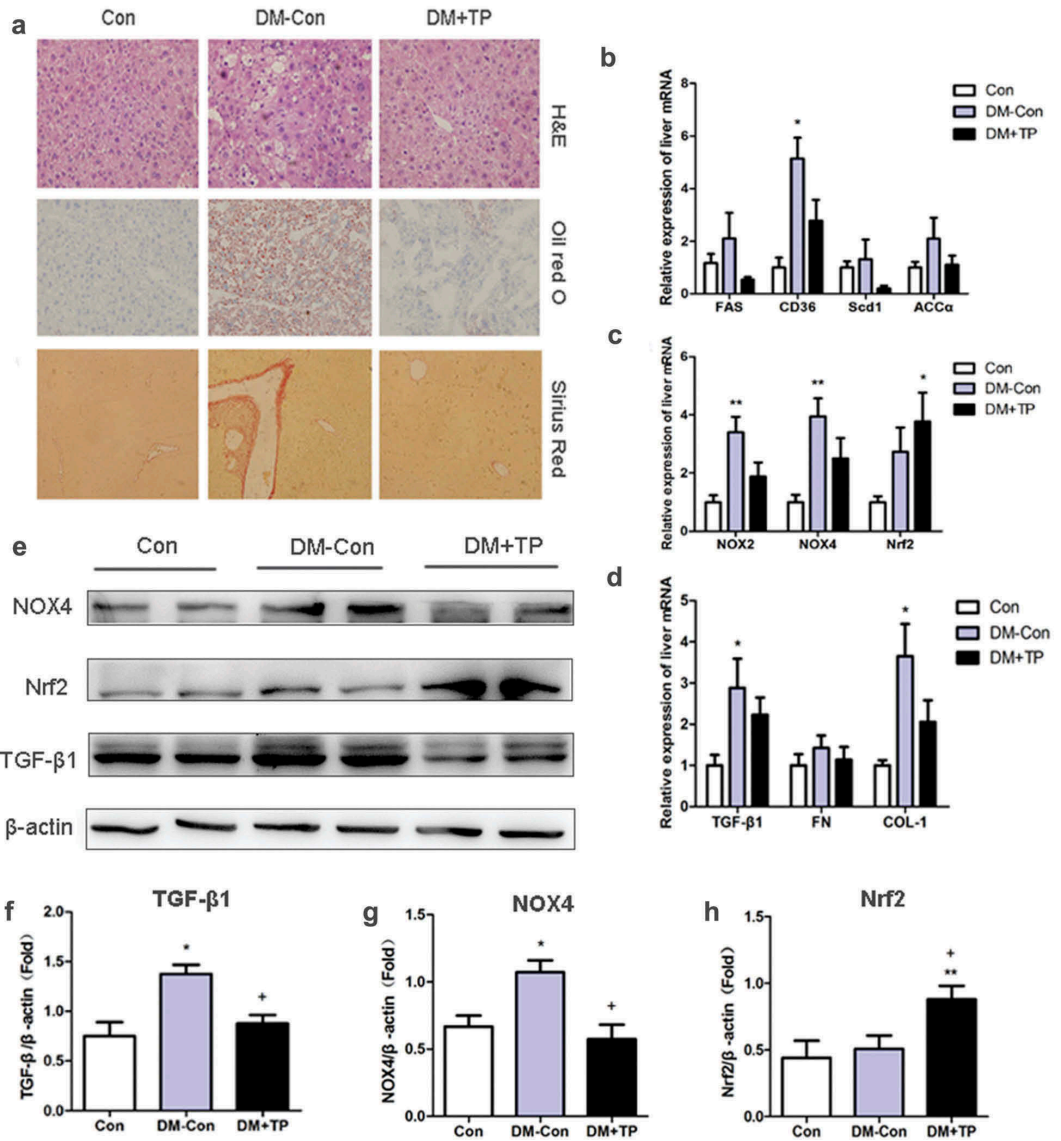
(a) Liver histologic changes in each groups. Representative images of hematoxylin-eosin (H&E) staining, Oil red O staining and Sirius Red staining. (Original magnification 200×). (b-d) The changes in mRNA expression of lipid synthesis, oxidative and fibrosis-related genes of liver in each group after BAT transplantation (n = 5-8/group). (b) Relative mRNA expression of liver FAS, CD36, Scd1 and ACCα. (c) Relative mRNA expression of liver NOX2, NOX4and Nrf2. (d) Relative mRNA expression of liver TGF-β1, FN and COL-1. (e-h) Representative Western blot showing TGF-β1, Nrf2, Nox4 and β-actin and densitometric analysis of Western results. *P < 0.05 vs Con; **P < 0.01 vs Con; ^+^P < 0.05 vs DM-Con

In order to observe the pathological changes in the liver, we performed H&E, Oil Red O and Sirius Red staining. Hepatic lobules with unclear structure, hepatocytes with enlarged volume and obvious nucleus and cell gap with unclear boundaries were found in the liver tissues of DM-Con group mice from H&E staining. Severe lipid and collagen deposition was also found in them from Oil Red O and Sirius Red staining. But these changes were almost reversed after BAT transplantation (Figure 2(a)).

To investigate the effects of BAT transplantation on liver metabolism of diabetic mice, the mRNA of liver such as FAS, CD36, Scd1, ACCα, NOX2, NOX4, Nrf2, TGF-β1, FN and COL-1 were analysed by qRT-PCR. The mRNA of CD36, NOX2, NOX4, TGF-β1 and COL-1 was significantly up-regulated in DM-Con group mice compared with those in Con group, whereas there was no significant difference of the expression of these genes after BAT transplantation. Strikingly, the expression of the other genes such as FAS, Scd1, ACCα and FN had no difference between three groups. However, the expression of antioxidant stress index Nrf2 was significantly up-regulated after BAT transplantation, indicating the ability to resist oxidative stress of liver might be improved (Figure 2(b–d)). The results of western blot showed that the protein expression of TGF-β1 and NOX4 was significantly up-regulated in DM-Con group mice compared with those in the Control group and significantly down-regulated after BAT transplantation compared with DM-Con group. The results of Nrf2 protein expression were the same as the mRNA result (Figure 2(e–h)).

These data showed that liver of diabetic mice suffering from severe lipid deposition, oxidative stress and fibrosis. But BAT transplantation can ameliorate the liver metabolism and reverse hepatic pathological changes in diabetic mice.

### BAT transplantation increases circulating miR-99a in diabetic mice

**Figure 3.**
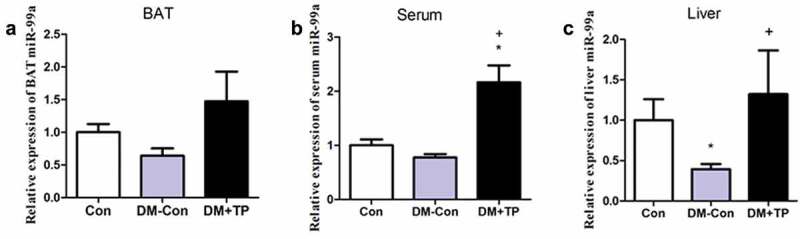
The changes in the expression of miR-99a in BAT (a), serum (b) and liver (c) in each group after BAT transplantation (n = 5–6/group). *P < 0.05 vs Con; ^+^P < 0.05 vs DM-Con

Since BAT is the main resource of miRNAs, we determined whether BAT transplantation can increase circulating miR-99a in diabetic mice by detecting the expression of miR-99a in BAT, serum and liver tissue using qRT-PCR. As shown in Figure 3, miR-99a levels of BAT, serum and liver in DM-Con group mice all decreased compared with Con group mice. BAT transplantation can up-regulate the expression level of BAT miR-99a compared with that of DM-Con group, but the difference was not statistically significant (P > 0.05) (Figure 3(a)). As expected, BAT transplantation significantly up-regulated serum and liver miR-99a expression levels (Figure 3(b,c)). These data showed that BAT transplantation can increase circulating miR-99a levels in diabetic mice.

### miR-99a regulates oxidative stress of LO2 cells by targeting inhibition of NOX4

**Figure 4.**
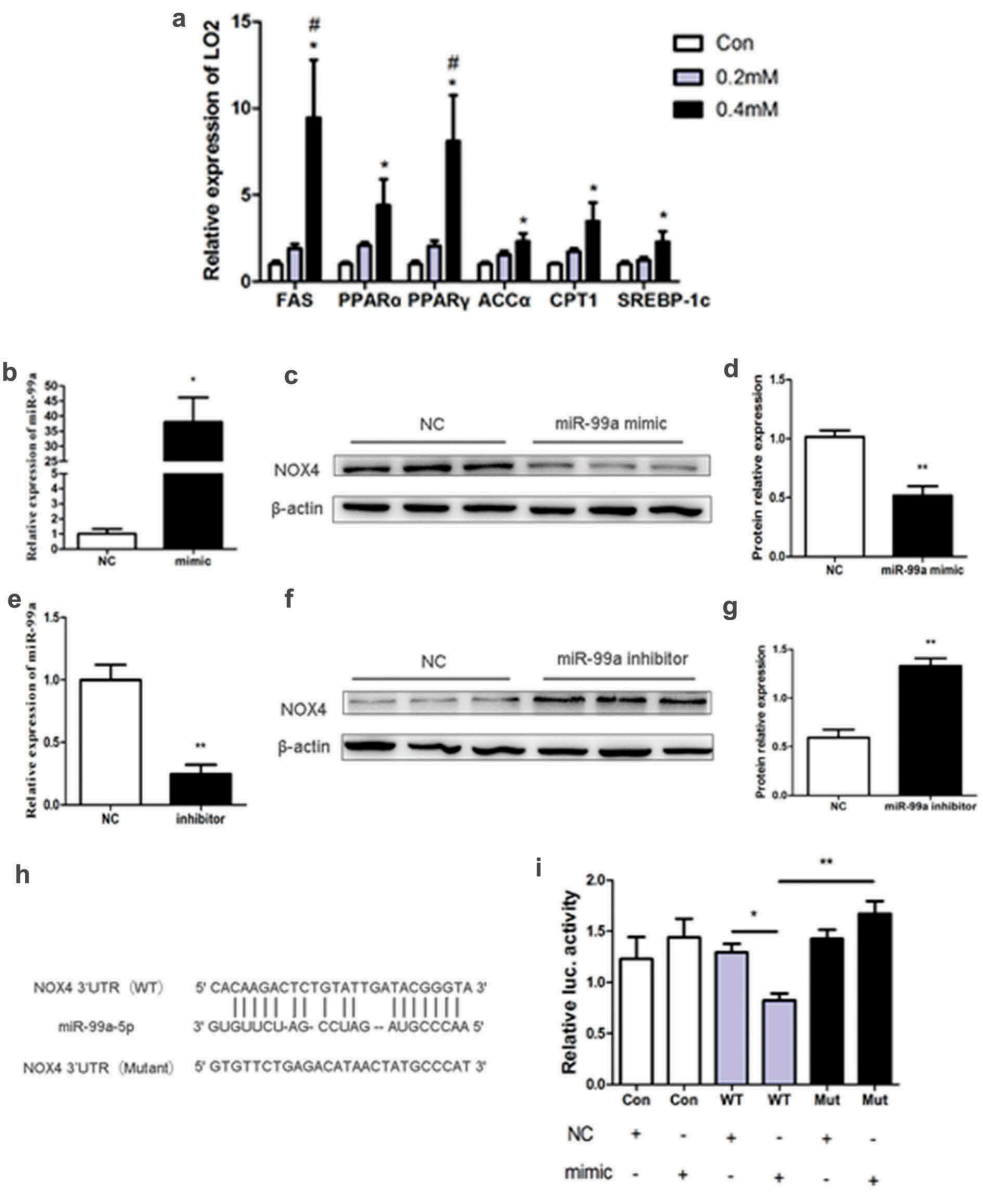
(a) Effects of different PA concentrations on mRNA expression of lipid metabolism-related gene in LO2 cells (n = 4/group). (b-d) The expression of miR-99a (b), NOX4 protein (c) and its quantitative analysis (d) in LO2 cells after transfected with miR-99a mimic (n = 3-4/group). (e-g) The expression of miR-99a (e), NOX4 protein (f) and its quantitative analysis (g) in LO2 cells after transfected with miR-99a inhibitor (n = 3-4/group). (h) Schematic diagram of binding sites of miR-99a-5p with NOX4 3ʹUTR. (i) Luciferase activity of LO2 cells after cotransfection with miR-99a mimic and pMIR-REPORT-NOX4 (n = 3-4/group). *P< 0.05; **P< 0.01. ^#^*P*< 0.05 vs 0.2mM. WT: Wild-type; Mut: Mutant-type

In vitro, we used different concentrations of PA to treat LO2 cells. The mRNA expression of liver lipid metabolism-related genes such as FAS, PPARα, PPARγ, AAAα, CPT1 and SREBP-1c was significantly up-regulated in LO2 cells only with treatment of 0.4 mM PA (Figure 4(a)). Therefore, we chose 0.4mM of PA as the suitable stimulation concentration.

In order to verify whether miR-99a improves hepatocyte oxidative stress by targeting inhibition of NOX4, we transfected LO2 cells with miR-99a mimic or inhibitor and then evaluated the effects using qRT-PCR and western blot. The expression of miR-99a was significantly up-regulated and the expression of NOX4 protein was significantly down-regulated after transfection with miR-99a mimic (Figure 4(b–d)). Conversely, LO2 cells transfected with miR-99a inhibitor resulted in significant inhibition of miR-99a expression and significant up-regulated NOX4 protein expression (Figure 4(e–g)).

NOX4 and miR-99a-5p were predicted by online prediction site TargetScan (http://www.targetscan.org/vert_72/), and it was found that NOX4 3ʹUTR contained the binding site of miR-99a-5p (Figure 4(h)). Therefore, we constructed dual-luciferase reporter gene containing two binding sites (wild type and mutant type) for miR-99a. As shown in (Figure 4(i)), co-transfection of wild-type NOX4 3ʹUTR plasmid and miR-99a mimic significantly down-regulated luciferase activity. By contrast, co-transfection of mutant NOX4 3ʹUTR plasmid with miR-99a mimic did not down-regulate luciferase activity.

Altogether, these data showed us that miR-99a can improve the oxidative stress of LO2 cells by targeting the 3ʹUTR of NOX4 mRNA.

## Discussion

Type 2 diabetes is a growing global health problem and its main features are insulin resistance and relative deficiency of insulin. Insulin resistance (IR) causes hyperglycaemia and reactive hyperinsulinemia, which in turn promotes lipid accumulation and ultimately affects lipid metabolism in the liver [21]. The liver is also the main target organ for insulin regulation of glycogen storage and gluconeogenesis. Therefore, chronic liver disease is an important complication of type 2 diabetes, and the risk of death from it is significantly increased in patients with type 2 diabetes [22].

NAFLD is a chronic liver disease of liver steatosis that affects the health of the majority of the world’s population as the economy develops. Pathological changes in NAFLD can range from simple hepatic steatosis (HS) to NASH, leading to liver fibrosis and cirrhosis. However, little is known about the pathogenesis of NAFLD nowaday. The more commonly recognized ‘second hit theory’ was first proposed by Day and James [23]. Liver TG accumulation and oxidative stress are the main features of the two strikes, respectively [24]. In recent years, this theory has gradually been replaced by ‘multiple-hit model’ [5], but oxidative stress is still crucial in the pathogenesis of NAFLD. Nicotinamide adenine dinucleotide phosphate (NADPH) oxidases (NOXs) are the main source of reactive oxygen species (ROS) and NOX1, NOX2 and NOX4 are three main subtypes expressed in the liver [25]. NOX4 plays a more important role in liver damage and fibrosis [26,27] and promotes the synthesis of collagen in the signalling of TGF-β1 [28]. Diabetes is a major risk factor for liver fibrosis and cirrhosis in NASH because persistent hyperglycaemia stimulates hepatic stellate cell (HSC) proliferation and produces extracellular matrix by activating NOXs [29].

In the present study, a mouse model of type 2 diabetes was established with HFD combined with STZ. In this non-genetic rodent model, β-cell mass loss and/or dysfunction by STZ insult are potentiated by peripheral insulin resistance or inflammation response induced by HFD. The HFD/STZ mouse model shows a similar pattern to that of type 2 diabetic patients [19,30–32]. Serum TG and LDL-C in DM group were higher than those in Con mice. The expression of lipid synthesis, oxidative stress and fibrosis-related genes in the liver was higher than those in the Con group. Pathological changes also suggested that liver lipids and collagen deposits were obvious. Therefore, it was considered that lipid tissue deposition, oxidative stress and fibrosis were observed in liver tissue. Therefore, it was considered that the changes of lipid deposition, oxidative stress and fibrosis were obvious in the liver of diabetic mice.

In the past few years, BAT was found in adults and mainly located on the collarbone and neck area [33,34]. However, BAT activity is associated with energy metabolism and is significantly reduced in overweight or obese individuals. This experiment mainly explored the effect of BAT transplantation on liver metabolism in diabetic mice, so BAT transplantation and sham operation intervention were only performed in diabetic mice. In this study, BAT transplantation significantly decreased the blood glucose in diabetic mice as reported in previous studies [35,36], although not to completely normal range. However, it was found that serum TG and LDL-C were significantly decreased in diabetic mice after BAT transplantation. In addition, BAT transplantation significantly down-regulated liver genes expression related to lipid metabolism, oxidative stress and fibrosis including FAS, CD36, Scd1, ACCα, NOX2, NOX4, TGF-β1, FN and COL-1 compared with DM-Con group and up-regulated antioxidant gene Nrf2. In general, in order to maintain the balance, oxygen-free radicals are cleared by the powerful antioxidant system in the liver. Sustained hyperglycaemia can stimulate the proliferation of hepatic stellate cells by activating NOXs and produce extracellular matrices [37]. Therefore, to some extent, the antioxidant levels of diabetic mice could be elevated but still not enough to combat oxidative stress induced by diabetes. In addition, under the microscope, the lipid and collagen deposition in the liver tissue of the BAT transplantation group was almost reversed. The above results indicated that BAT transplantation can improve liver lipid metabolism, oxidative stress and fibrosis in diabetic mice.

Recent studies suggest that adipose tissue can regulate liver metabolism by secreting various adipokines, including miRNAs [13]. miRNAs could negatively regulate the transcription of target genes [38], which were shown that played an important role in metabolic syndrome, especially related to the liver [39]. Studies had shown that specific miRNA expression profile was closely related to NAFLD [40–42]. Our previous study had also shown that activation of brown adipose tissue could significantly up-regulate the levels of serum miR-99a, miR-99b, miR-30b, miR-100, etc. [43]. Study by Thomou et al. has shown that adipose tissue was the main source of circulating miRNAs and that the level of miR-99a and miR-99b in the circulation was significantly increased after BAT transplantation in a fat-specific knockout of the miRNA-processing enzyme Dicer mice [13]. And miR-99b regulates liver metabolism by targeting FGF21. However, the specific role of miR-99a in liver metabolism remains unclear. In NAFLD rat model, miR-99a was significantly down-regulated [44]. And miR-99a played a role in cell invasion and migration by targeting NOX4 [45]. This suggests that miR-99a may be involved in the pathogenesis of NAFLD through NOX4. In our previous study, the results showed that BAT transplantation can significantly increase the expression of endogenous BAT UCP1 mRNA and protein. And HE staining showed the brown adipocytes’ volume in the BAT of the DM-Con group was increased, the nucleus was located at the edge of the cell, and the lipid droplets were large and single, that is, the phenomenon of ‘whitening’ appeared. But BAT morphology was significantly improved in diabetic mice after BAT transplantation. In the present study, miR-99a expression in the liver significantly decreased in diabetic mice and significantly increased in both liver and serum after BAT transplantation. In *vitro*, LO2 cells incubated with 0.4 mM PA was used to mimic fatty liver disease as reported before [46,47]. The expression of NOX4 protein was significantly decreased after transfection with miR-99a mimic, which could be reversed by transfection with miR-99a inhibitor. Furthermore, luciferase reporter gene experiments confirmed that miR-99a targets and regulates the expression of NOX4. There were also many in vitro studies on miRNAs involved in the regulation of lipid metabolism, such as miR-146a-5p [48], miR-125a-5p [49] and so on. Therefore, there might be multiple miRNAs involved in the regulation of hepatic lipid metabolism, and our findings could provide some help for the treatment of NAFLD.

However, there were still some limitations in this study. Firstly, we did not detect the interscapular BAT temperature, which represents the thermal effect of BAT. Secondly, this study did not correct other functional indicators of oxidative stress (e.g. ROS level) or signalling components downstream of NOX4 relevant to HSC activation. Finally, in order to further verify the experimental results of this study, we still need to complete the in vivo experiment of knockout or overexpression model. Therefore, we still need to carry out relevant experiments to improve the research in the future.

In conclusion, BAT transplantation can improve liver lipid metabolism, oxidative stress and fibrosis in diabetic mice partially through increasing circulating miR-99a, which in turn negatively regulating NOX4 in the liver.
